# Generation of a Genetically Modified Chimeric *Plasmodium falciparum* Parasite Expressing *Plasmodium vivax* Circumsporozoite Protein for Malaria Vaccine Development

**DOI:** 10.3389/fcimb.2020.591046

**Published:** 2020-12-17

**Authors:** Yukiko Miyazaki, Catherin Marin-Mogollon, Takashi Imai, António M. Mendes, Rianne van der Laak, Angelika Sturm, Fiona J. A. Geurten, Shinya Miyazaki, Severine Chevalley-Maurel, Jai Ramesar, Surendra K. Kolli, Hans Kroeze, Roos van Schuijlenburg, Ahmed M. Salman, Brandon K. Wilder, Arturo Reyes-Sandoval, Koen J. Dechering, Miguel Prudêncio, Chris J. Janse, Shahid M. Khan, Blandine Franke-Fayard

**Affiliations:** ^1^ Department of Parasitology, Leiden University Medical Center, Leiden, Netherlands; ^2^ Department of Infectious Diseases and Host Defense, Gunma University Graduate School of Medicine, Maebashi, Japan; ^3^ Instituto de Medicina Molecular João Lobo Antunes, Faculdade de Medicina, Universidade de Lisboa, Lisboa, Portugal; ^4^ TropIQ Health Sciences, Nijmegen, Netherlands; ^5^ The Jenner Institute, Nuffield Department of Medicine, University of Oxford, Oxford, United Kingdom; ^6^ Vaccine and Gene Therapy Institute, Oregon Health and Science University, Portland, Oregon, United States

**Keywords:** malaria, *Plasmodium falciparum*, *Plasmodium vivax*, circumsporozoite protein (CSP), chimeric parasites, vaccines

## Abstract

Chimeric rodent malaria parasites with the endogenous circumsporozoite protein (*csp*) gene replaced with *csp* from the human parasites *Plasmodium falciparum* (*Pf*) and *P. vivax* (*Pv*) are used in preclinical evaluation of CSP vaccines. Chimeric rodent parasites expressing *Pf*CSP have also been assessed as whole sporozoite (WSP) vaccines. Comparable chimeric *P. falciparum* parasites expressing CSP of *P. vivax* could be used both for clinical evaluation of vaccines targeting *Pv*CSP in controlled human *P. falciparum* infections and in WSP vaccines targeting *P. vivax* and *P. falciparum*. We generated chimeric *P. falciparum* parasites expressing both *Pf*CSP and *Pv*CSP. These *Pf*-*Pv*CSP parasites produced sporozoite comparable to wild type *P. falciparum* parasites and expressed *Pf*CSP and *Pv*CSP on the sporozoite surface. *Pf*-*Pv*CSP sporozoites infected human hepatocytes and induced antibodies to the repeats of both *Pf*CSP and *Pv*CSP after immunization of mice. These results support the use of *Pf*-*Pv*CSP sporozoites in studies optimizing vaccines targeting *Pv*CSP.

## Introduction

*Plasmodium falciparum (Pf)* and *P. vivax* (*Pv*) are two major human malaria parasites that threaten public health, mainly in tropical areas. Malaria parasites are transmitted by *Anopheles* mosquitoes where they develop into infectious sporozoites that reside in the salivary glands. After a bite of an infected mosquito, sporozoites enter the blood stream of the mammalian host and migrate to the liver where they invade hepatocytes. Inside hepatocytes, parasites replicate to form thousands of daughter merozoites, which are released into the blood and invade erythrocytes. The pre-erythrocytic stages of *Plasmodium* parasites, i.e. sporozoites and liver stages, are attractive targets of leading malaria vaccine candidates, including RTS,S, the most advanced subunit vaccine against *P. falciparum* tested in Phase III clinical trials (Kester et al., 2009; Cohen et al., 2010; Agnandji et al., 2012; Olotu et al., 2016). RTS,S is based on the circumsporozoite protein (CSP), a major surface protein expressed by *Plasmodium* sporozoites and early liver stages which plays a critical role in sporozoite formation, invasion of mosquito salivary glands and invasion of host hepatocytes (Ménard, 2000; Sinnis and Coppi, 2007; Coppi et al., 2011). This protein contains a highly conserved central repeat region flanked by N- and C -terminal regions. B-cell responses to *Pf*CSP target predominantly the protein’s central NANP repeat region, generating antibodies capable of protecting animal models from *Plasmodium* infection and NANP antibodies induced by RTS,S immunization are associated with clinical protection (White et al., 2013; Foquet et al., 2014; Sack et al., 2014; Triller et al., 2017). CSP from *P. vivax* also contains an immunogenic central repeat region with two major *Pv*CSP alleles, VK210 and VK247 considered as important targets for a vaccine (Yadava and Waters, 2017). So far pre-erythrocytic subunit vaccines targeting CSP, including RTS,S, have shown low to modest protective efficacy in clinical trials and in field studies (Agnandji et al., 2015; Hoffman et al., 2015; Clemens and Moorthy, 2016; Long and Zavala, 2016; Olotu et al., 2016; Healer et al., 2017; Mahmoudi and Keshavarz, 2017) emphasizing the need to improve CSP-based vaccination approaches which requires efficient preclinical and clinical evaluation of different vaccines. Clinical evaluation of *P. falciparum* vaccines is greatly aided by the ability to vaccinate individuals and then examine vaccine efficacy in controlled human malaria infections (CHMI) (Sauerwein et al., 2011; Spring et al., 2014; Bijker et al., 2016; Stanisic et al., 2018). Although CHMI for *P. vivax* has been developed (Arévalo-Herrera et al., 2016; Bennett et al., 2016; Payne et al., 2017; Hall et al., 2019), the lack of a continuous *in vitro* culture system for *P. vivax* blood stages limits the availability of *P. vivax* sporozoites for clinical human infections. In addition, since *P. vivax* forms dormant hypnozoites in the liver, which can causing infection relapses, safe and effective means for clearance of hypnozoites are essential for *P. vivax* CHMI studies (Payne et al., 2017).

In multiple preclinical studies, the evaluation of the efficacy of CSP-based vaccines and anti-CSP antibodies has been performed using chimeric rodent malaria parasites expressing CSP from either *P. falciparum* or *P. vivax* (Espinosa et al., 2013; Gimenez et al., 2017; Salman et al., 2017; Vijayan et al., 2017; de Camargo et al., 2018; Marques et al., 2020). The availability of comparable chimeric *P. falciparum* parasites expressing *Pv*CSP would permit the analysis of protective efficacy of *Pv*CSP-based vaccines in clinical studies using *P. falciparum*-based CHMI, bypassing the need for *P. vivax* sporozoite production and hypnozoite elimination. However, in contrast to efficient *Pv*CSP expression and sporozoite production in chimeric rodent parasites, we found that chimeric *P. falciparum* lines with the *Pf*CSP gene replaced by *Pv*CSP, failed to produce salivary gland sporozoites, indicating that *Pv*CSP cannot fully complement the role of *Pf*CSP in *P. falciparum* sporozoite formation (Marin-Mogollon et al., 2018). To develop chimeric *P. falciparum* sporozoites expressing *Pv*CSP, we therefore aimed at generating a chimeric *P. falciparum* line expressing both the endogenous *Pf*CSP and an engineered *Pv*CSP. It has been shown that introducing the *P. falciparum csp* gene as an additional copy into the genome of the rodent parasite *P. berghei* (*Pb*) results in development of viable and infective sporozoites, expressing both *Pb*CSP and *Pf*CSP in pre-erythrocytic stages (Mendes et al., 2018a; Mendes et al., 2018b; Reuling et al., 2020). We used CRISPR/Cas9 gene editing to introduce a chimeric *Pvcsp* gene, containing repeats of both the VK210 and VK247 alleles, into the *Pfp47* locus of the *P. falciparum* NF54 genome. We found that these *Pf*-*Pv*CSP chimeric *P. falciparum* parasites produced viable, motile sporozoites expressing both *Pf*CSP and chimeric *Pv*CSP at the sporozoite surface that are infectious to primary human hepatocytes. Immunization of mice with these sporozoites resulted in induction of antibodies against the repeats of both *Pf*CSP and chimeric *Pv*CSP. These results not only show promise for the use of *Pf*-*Pv*CSP sporozoites in clinical CHMI studies for optimization of vaccines targeting *Pv*CSP, but also suggest that such chimeric sporozoites can be used in whole, attenuated sporozoite vaccination approaches, capable of inducing cross-protective immune responses against both *P. falciparum* and *P. vivax*.

## Materials and Methods

### *In Vitro* Cultivation of *P. falciparum* Blood Stages

Wild type *P. falciparum* NF54 (WT NF54) parasites (Ponnudurai et al., 1981) were obtained from the Radboud University Medical Center (Nijmegen, The Netherlands). Parasites were cultured in RPMI-1640 culture medium supplemented with L-glutamine and 25mM HEPES (Gibco Life Technologies), 50 mg/L hypoxanthine (Sigma), 0.225% NaHCO_3_ and 10% human serum at a 5% hematocrit under 4% O_2_, 3% CO_2_ and 93% N_2_ gas-conditions at 75 rpm at 37°C in a semi-automated culture system (Infers HT Multitron and Watson Marlow 520U) as previously described (Mogollon et al., 2016). Fresh human serum and human red blood cells (RBC) were obtained from the Dutch National Blood Bank (Sanquin Amsterdam, the Netherlands; permission granted from donors for the use of blood products for malaria research and microbiology tested for safety). Production of genetically modified parasites and characterization of these parasites throughout their life cycle, including mosquito transmission, was performed under GMO permits IG 17-230_II-k and IG 17-135_III.

### Laboratory Animals and Ethics Statement

Female C57BL/6Jico mice (6–7 weeks; Charles River, NL) were used. All animal experiments were granted a licence by the Competent Authority after an advice on the ethical evaluation by the Animal Experiments Committee Leiden (AVD1160020171625). All experiments were performed in accordance with the Experiments on Animals Act (Wod, 2014), the applicable legislation in the Netherlands and in accordance with the European guidelines (EU directive no. 2010/63/EU) regarding the protection of animals used for scientific purposes. All experiments were performed in a licenced establishment for the use of experimental animals (LUMC). Mice were housed in individually ventilated cages furnished with autoclaved aspen woodchip, fun tunnel, wood chew block and nestlets at 21 ± 2°C under a 12:12 h light-dark cycle at a relative humidity of 55 ± 10%.

### Anopheles Rearing

Mosquitoes from a colony of *Anopheles stephensi* (line Nijmegen SDA500) were used. Larval stages were reared in water trays at a temperature of 28 ± 1°C and a relative humidity of 80%. Adult females were transferred to incubators with a temperature of 28 ± 0.2°C and a relative humidity of 80%. For the transmission experiments, 3- to 5-day-old mosquitoes were used.

### Generation of the Chimeric Pf-PvCSP Parasites

Chimeric *P. falciparum* parasites expressing both *Pf*CSP and chimeric *Pv*CSP (*Pv*CSP-chi), were generated using the previously described pLf0019 construct (Mogollon et al., 2016)(**Figure S1**), containing the *cas9* gene, together with the gRNA-donor DNA containing plasmid, pLf0109, targeting *Pfp47* which contains the *Pvcsp-chi* expression cassette. The pLf0019 construct contains a blasticidin (*bsd*) drug-selectable marker cassette and the pLf0109 gRNA-donor DNA construct contains a *hdhfr-yfcu* drug-selectable marker cassette for selection with WR99210 and 5-fluorocytocine (5-FC)(**Figure S1**). To generate pLf0109, the plasmid pLf0047 (Marin-Mogollon et al., 2019), containing two homology regions and a gRNA expression cassette targeting *Pf*p47 (PF3D7_1346800), was modified by introducing a *Pvcsp-chi* expression cassette, which was commercially synthesized (Integrated DNA Technologies, Inc.; see **Figure S2**). This *Pvcsp-chi* expression cassette contains a codon-optimized *Pvcsp* open reading frame (ORF) containing the N- and C-terminal regions of the *Pv*csp VK210 allele that flank a chimeric repeat sequence comprising repeats of both the VK210 allele (three times the repeat GDRADGQPA/GDRAAGQPA) and the VK247 allele (three times the repeat ANGAGNQPG/ANGAGDQPG). The ORF of *Pvcsp-chi* is flanked by *Eco*RV/*Eco*RI restriction sites and cloned into pLf0040 (cut *Eco*RV/*Eco*RI) (Marin-Mogollon et al., 2018) which contains 967bp of the promoter regions of *Pfcsp* (PF3D7_0304600) and 917bp of 3’UTR of *Pfcsp* resulting in intermediate plasmid T24 (**Figure S1**). This plasmid was digested with *Apa*I/*Sac*II to obtain the complete *Pv*csp-*chi* expression cassette containing the *Pf*csp promoter, the *Pvcsp-chi* ORF with the chimeric VK210/VK247 repeats, and the *Pf*csp 3’UTR. This expression cassette was inserted into vector pLf0047 (**Figure S1**) using the same restriction enzymes (*Apa*I/*Sac*II) resulting in the final construct pLf0109. Plasmids for transfection were isolated from 250 ml cultures of *Escherichia coli* XL10-Gold Ultracompetent Cells (Stratagene, NL) by maxi-prep (PureYield™ Plasmid Maxiprep System (Promega, NL) to generate 25–50 µg of DNA used per transfection. Transfection of WT *Pf*NF54 parasites was performed using the ‘spontaneous uptake method’ as previously described (Miyazaki et al., 2020). Briefly uninfected RBC (300 μl of packed RBCs) were transfected with the constructs pLf0019 and pLf0109 (a mixture of 25 μg of each circular plasmid in 200 μl cytomix) using the Gene Pulser Xcell electroporator (BioRad) with a single pulse (310 V, 950 μF). Subsequently, the transfected RBCs were washed with complete RPMI1640 medium and mixed with WT *Pf*NF54 infected RBC (iRBC) with a parasitemia of 0.5% and a hematocrit of 5%. These cells were transferred into a 10 ml culture flask and the cultures were maintained under standard conditions in the semi-automated culture system (Mogollon et al., 2016). Selection of transfected parasites was performed by applying double-positive drug pressure from day 3 after transfection using the drugs WR99210 (2.6 nM) and BSD (5 µg/ml). The positive drug pressure was maintained until parasites were detectable in thin blood-smears (at day 15 after transfection). Subsequently, the parasites were maintained in drug-free medium for 2–4 days until the parasitemia reached over 10%, followed by applying negative selection by addition of 5-FC (1 µM) as described before (Mogollon et al., 2016) in order to eliminate parasites that retained the donor DNA construct as episomal plasmid and to enrich for the transfected parasites containing the donor DNA integrated into the genome. Negative drug pressure in the cultures was maintained until thin blood-smears were parasite-positive (after 7 days). After the negative selection, iRBC were harvested from cultures for genotyping by diagnostic PCR and Southern blot analysis. Subsequently, selected parasites were cloned by limiting dilution as previously described (Marin-Mogollon et al., 2018). Briefly, serial dilutions were performed with uninfected RBC in complete medium (1% hematocrit and 20% serum) and cultured in a total volume of 100 µl incubated in 96-well plates, resulting in 8 rows with the following numbers of iRBC per well: 100, 10, 5, 2.5, 1.25, 0.6, 0.3, 0.15. Plates were incubated in a Candle Jar at 37°C and culture medium was changed every other day. Every 5 days RBC were added resulting in an increase of the hematocrit from 1% to 5%. At days 10 to 11, samples were collected for thick smear analysis from the rows with the highest numbers (100 and 10) of iRBC/well. At day 21 thick smears were made from samples from all rows. Clones were selected from dilutions/row with less than 40% of the wells being parasite-positive. These clones were transferred in 10 ml culture flasks at 5% hematocrit under standard culture conditions in the semi-automated culture system for collection of parasites for further genotype and phenotype analyses (see next section).

### Genotyping of Pf-PvCSP Parasites

Diagnostic PCR and Southern blot analysis of digested DNA were performed from material isolated from iRBC obtained from 10 ml cultures, pelleted by centrifugation (1500 g, 5 min). RBC were then lysed with 5 ml of cold (4°C) erythrocyte lysis buffer (10× stock solution 1.5 M NH_4_Cl, 0.1 M KHCO_3_, 0.01 M Na_2_EDTA; pH 7.4; (Janse et al., 2006)) and parasites were treated with proteinase-K before DNA isolation by standard phenol-chloroform methods. Correct integration of the donor construct was analyzed by long-range PCR (LR-PCR) (primers P1/P3) and standard PCR amplification of the *Pvcsp-chi* gene (primers P4/P5), the *Pfp47* gene (primers P6/P7) and the fragment for 5’ integration (5’int; primers P1/P2). The PCR fragments were amplified using KOD Hot start polymerase following standard conditions with an annealing temperature of 52°C for 15 s, 55°C for 15 s, and 63°C for 25 s and an elongation step of 68°C for 4 min for LR-PCR and with an annealing temperature of 50°C for 10 s, 55°C for 10 s, and 60°C for 10 s and an elongation step of 68°C for 1 min for the other PCRs. Southern blot analysis was performed with genomic DNA digested with *Hpa*I and *Eco*RI (4 h at 37°C) to confirm integration of the replacement of the *Pfp47* gene by the *Pvcsp-chi* expression cassette. Digested DNA was hybridized with probes targeting the *Pfp47* homology region 1 (HR1), amplified from WT *Pf*NF54 genomic DNA by PCR using the primers P8/P9 and a second probe targeting ampicillin (Amp) gene, amplified from plasmid pLf0109 by PCR using the primers P10/P11.

#### Phenotype analysis Pf-PvCSP parasites: blood stages, gametocytes, oocysts, and sporozoites

The growth rate of asexual blood stages of the *Pf*-*Pv*CSP was determined in 10 ml cultures maintained in the semi-automated culture system under standard culture conditions. Briefly, a culture with 0.1% parasitemia was established in complete culture medium at a hematocrit of 5%. Medium was changed twice daily and the culture was maintained for a period of 3 days without refreshing RBC. For determination of the course of parasitemia, triplicate samples of 100 µl were collected daily from all cultures and cells pelleted by centrifugation (9485 g; 30 s) and stained with Giemsa. *P. falciparum* gametocytes cultures were generated using standard culture conditions as previously described (Marin-Mogollon et al., 2018) with some modifications. Briefly, parasites from asexual stage cultures were diluted to a final parasitemia of 0.5%, and cultures were followed during 14 days without adding new RBC. At day 14, gametocyte development was analyzed by counting male/female gametocytes (stage V) in Giemsa stained blood smears. To assess exflagellation, 20 µl samples of the *P. falciparum* gametocyte cultures at day 14 were diluted 1:1 with Fetal Calf Serum (FCS) at room temperature and exflagellation centers were examined and quantified 10–20 min after activation in a Bürker cell counter.

For analysis of oocyst and sporozoite production, *A. stephensi* mosquitoes were fed when cultures contained mature stage V gametocytes and showed more than 80 exflagellating parasites per 10^5^ erythrocytes. Mosquito feeding was performed using the standard membrane feeding assay (SMFA) (Ponnudurai et al., 1989). Oocysts were analyzed between day 10 and 12 and salivary gland sporozoites were counted at day 18 and 21 after feeding. Oocyst were counted in 10–30 midguts, dissected in a droplet of mercurochrome (1%) in distilled water on a glass slide. Midguts were covered with a coverslip and oocyst images were recorded from dissected midgut in PBS 1 X (100× magnification). For counting sporozoites, salivary glands from 30–60 mosquitoes were dissected and homogenized using a grinder in 100 µl of RPMI pH 7.2 and sporozoites were analyzed in a Bürker cell counter using phase-contrast microscopy.

### Analysis of PvCSP-chi and PfCSP Expression in Sporozoites

To analyze CSP expression in fixed *Pf*-*Pv*CSP and WT *Pf*NF54 salivary gland sporozoites by indirect immunofluorescence assay (IFA), samples of purified salivary gland sporozoites (20 µl) were placed on a 8-well black cell-line diagnostic microscope slide (Thermo Scientific, NL), dried for 10 min, and fixed with 4% paraformaldehyde for 30 min at room temperature. After fixation the slides were washed three times with PBS and permeabilized with 20 µl of 0.5% triton in PBS and then blocked with 10% of FCS in PBS for 1h. Fixed cells were washed with PBS and incubated overnight at 4°C with the following monoclonal CSP antibodies: mouse anti-*Pf*CSP (mAb 2A10, MRA-183; 1:200 dilution of 1 mg/ml stock solution); mouse anti-*Pv*CSP VK210 (mAb 2F2; MRA-184; (Salman et al., 2017); 1:200 dilution of 109 µg/ml stock solution) and mouse anti-*Pv*CSP VK247 (mAb 2E10.E9; MRA-185; (Salman et al., 2017); 1:200 dilution of 125 µg/ml stock solution. Subsequently, cells were rinsed three times with PBS and incubated with the secondary antibodies Alexa FLuor^®^488/594-conjugated chicken anti-mouse (Invitrogen Detection technologies, NL at 1:500 dilution). Finally, the cells were washed again three times with PBS and stained with the DNA-specific dye Hoechst-33342 at a final concentration of 10 µM. Fixed cells were covered with 1–2 drops of an anti-fading agent (Image-iT™ FX, Life technologies), and a coverslip placed on the cells and sealed with nail polish. Stained cells were analyzed for fluorescence using a Leica fluorescence MDR microscope (100× magnification). Pictures were recorded with a DC500 digital camera microscope using Leica LAS X software with the following exposure times: Alexa 488: 1.0 s; Alexa 594: 0.6 s; Hoechst 0.2 s; bright field 0.62 s (1× gain) or with a Leica SP8 upright confocal microscope.

To analyze CSP expression of live *Pf*-*Pv*CSP and WT *Pf*NF54 salivary gland sporozoites by indirect immunofluorescence assay (IFA), samples of 2 × 10^5^ salivary gland sporozoites were aliquoted in 1.5 ml Eppendorf tubes and were incubated with the corresponding antibody (mouse anti-*Pf*CSP, mouse anti-*Pv*CSP VK210 or mouse anti-*Pv*CSPVK247 (1:100 dilution) in 100 µl of 1% (v/v) FCS in PBS 1×, for 1 h on ice. Subsequently, sporozoites were rinsed with 1.5 ml of 1% (v/v) FCS in PBS and pelleted by centrifugation at 9300 g for 4 min at 4°C. The sporozoite pellet was then resuspended in 100 µl 1% FCS in PBS containing the secondary antibodies; anti–mouse IgG Alexa 488 (1:200) and incubated for 1 h on ice. Sporozoites were rinsed with 1.5 ml of 1% (v/v) FCS in PBS and pelleted by centrifugation at 9300 g for 4 min at 4°C, and the resulting pellet was resuspended in 40 µl of PBS. Of the cell suspension containing sporozoites 20 µl was placed per well of an 8-well black cell-line diagnostic microscope slide (Thermo Scientific), incubated 30 min at room temperature (RT), and fixed with 80 µl of 4% PFA in PBS 1× for 30 min at RT. Finally, the wells were washed twice with PBS and stained with 10 µM Hoechst 33342 in PBS for 15 min at RT. For microscopy analysis, the preparations were covered with 1–2 drops of an anti-fading agent (Image-iT™ FX, Life technologies), and a coverslip was placed onto the cells and sealed with nail polish. Stained cells were analyzed for fluorescence using a Leica fluorescence MDR microscope (100× magnification). Pictures were recorded with a DC500 digital camera microscope using Leica LAS X software with the following exposure times: Alexa 488: 0.7 s; Hoechst 0.136 s; bright field 0.62 s (1× gain).

### Sporozoite Gliding Assay

Sporozoite motility was determined using a sporozoite gliding assay (Stewart and Vanderberg, 1988; van Schaijk et al., 2008). Glass slides were placed in a 24 wells plate (Thermofisher, DK) and coated with an anti-*Pf*CSP monoclonal 3SP2 (courtesy of Dr. M. McCall, Radboudumc, The Netherlands; 5 μg/ml in PBS) using 200 µl per slide, and left overnight at RT. The next day freshly isolated WT *Pf*NF54 and *Pf*-*Pv*CSP sporozoites, collected in RPMI (Thermofisher, Gibco 42401-018) with 10% FCS (Capricornscientific, FBS-12A CP15-1439) at RT were added to anti-*Pf*CSP coated slides that were washed with PBS. Per slide 30.000 sporozoites were added (in 200 µl RPMI with 10% FCS) and these glass slides were incubated for 2.5 h at 37°C under 5% CO_2_ conditions. Slides were then washed three times with PBS and fixed with 4% PFA (Thermo Scientific, NL) for 20 min at RT. Then, the slides were washed three times with PBS and blocked with 1% BSA (Roche diagnostic, DE) in PBS for 15 min. After BSA blocking, the slides were washed three times with PBS, and the primary antibody anti-Pf-3SP2 or anti-*Pv*CSP-VK210 Mab (mAb 2F2) (10 µg/ml) was added and incubated for 45 min at RT. Slides were then washed three times with PBS, and incubated with a secondary antibody goat anti-mouse Alexa fluor 488 (Invitrogen Thermofisher, Oregon, A11001) Mab (diluted 500 times in PBS) for 45 min (RT, in the dark). Finally, slides were washed three times with PBS in the dark, air dried until the slides were almost dry and mounted with Coverslips (with Image iT FX signal Enhancer; Invitrogen Thermofisher, USA) and examined with a Leica DMR fluorescent microscope with standard GFP filter for the gliding trails at 63× magnification. The percentage of gliding was calculated in 8–10 fields, measuring the number of gliding trails for the WT *Pf*NF54 sporozoites in comparison with the gliding trails for the *Pf-Pv*CSP sporozoites. The mean and standard deviation were determined for the percentage of gliding trail sporozoites.

### *In Vitro* Infection of Primary Human Hepatocytes by Sporozoites

*In vitro* primary human hepatocyte maturation assay was performed as described in (Marin-Mogollon et al., 2019) with few adaptations. Cryopreserved primary human hepatocytes, obtained from Xenothech, were thawed according to the manufacturer and seeded at a density of 60,000 cells/well in a collagen-coated 96-well clear-bottomed black plate for 2 days. Medium was refreshed daily (hepatocyte medium: Williams’s E medium supplemented with 10% heat inactivated fetal bovine serum, 1% penicillin-streptomycin, 1% fungizone, 0.1 lU/ml insulin, 70 µM hydrocortisone). Per well, 7 x 10^4^ freshly dissected *Pf-Pv*CSP and WT *Pf*NF54 sporozoites were added to the hepatocyte monolayer. After a quick spin (10 min at 1900 g), the plate was incubated at 37°C under 5% CO2. The medium was replaced with fresh hepatocyte culture medium 3 h post-invasion, and daily for 4 days thereafter. Then, hepatocytes were fixed with ice-cold methanol for at least 10 min on ice. A standard IFA was performed using an anti-*Pf*HSP70 primary antibody (StressMarq) with an Alexa fluor 594 (Invitrogen) secondary antibody and DAPI for nuclear staining. High content imaging was performed, using the ImageXpress Pico system (Molecular Devices). Number of parasites, number of hepatocytes and size of parasites were determined using the CRX software (Molecular Devices) and data analyzed using the GraphPad software.

### Rodent Immunization With Sporozoites and Antibody Production

For generation of sporozoite-extracts for immunization, salivary gland sporozoites were isolated as described in the previous sections. Aliquots of 10^5^ sporozoites in 50 µl PBS were made and stored at −80°C. After two rounds of freezing and thawing, 10^5^ sporozoites in 200 µl PBS were used to immunize mice by intravenous injection. Groups of six C57BL/6 mice were immunized three times with sporozoites at a 7-day interval, and blood was collected in heparinized capillaries from all mice by orbital vein puncture 1 day prior to the first and third immunization and one week after the last immunization. Plasma was collected after centrifugation of the blood samples (1500 g for 8 min at 4°C) and stored at −20°C until further analysis.

For assessment of anti-CSP antibody titers (total IgG), enzyme-linked immunosorbent assays (ELISAs) were performed. High protein-binding capacity 96-well ELISA plates (Nunc MaxiSorp™ flat-bottom) were coated with synthetic peptides (Sigma) based on the repeat sequences of *Pf*CSP and *Pv*CSP. Specifically, plates were coated with peptides with the amino acid sequence (NANP)4NVDPC for *Pf*CSP, the amino sequence GD RAD GQP AGD RAA GQP A for the *Pv*CSP VK210 and the amino sequence ANGAGNQPG ANGAGDQPG for the *Pv*CSP VK247. The plates were coated overnight at 4°C with a peptide concentration of 5 µg/ml in PBS with a volume of 50 µl per well. Plates were washed three times with 0.05% Tween20/BS (PBST) and blocked with 200 µl of PBST with 5% milk for 30 min at RT. Plates were then washed one additional time and plasma samples were diluted 1:50 in 1% milk PBST and a 3-point 1:3 dilution was carried out for each sample. After 3h incubation at RT, plates were washed three times with PBST and incubated for 1h at RT with the secondary antibody, horseradish peroxidase-labelled goat anti-mouse IgG (GE Healthcare UK) at a dilution of 1:2000. After six PBST washes, the reaction was developed by adding BD OptEIA™ TMB Substrate Reagent and incubating for 1 to 5 min at RT, before stopping the reaction by adding 50 µl Stop solution (2N H_2_SO_4_). Absorbance was immediately measured at 450 nm using a Tecan Infinite M200 plate reader. Data were analyzed in relation to a standard titration curve of at least 8 points starting a dilution of 1.5u/ml of the corresponding mouse monoclonal antibody: mouse anti-*Pf*CSP (mAb 2A10, MRA-183); mouse anti-*Pv*CSP VK210 (mAb 2F2; MRA-184) and mouse anti-*Pv*CSP VK247 (mAb 2E10.E9; MRA-185). All monoclonal antibodies were produced from hybridoma cell lines obtained from BEI Resources, NIAID, and kindly provided by Elizabeth Nardin.

### Statistics

Data were analyzed using GraphPad Prism software version 8.1.1 (GraphPad Software, Inc). Significance difference analyses between WT *Pf*NF54 and *Pf*-*Pv*CSP lines were performed using the unpaired Student’s t-test. The value of p < 0.05 was considered statistically significant (* p < 0.01, ** p < 0.001, *** p < 0.0001).

## Results

### Generation of Chimeric *Plasmodium falciparum* Sporozoites Expressing PfCSP and Chimeric PvCSP

**Figure 1:**
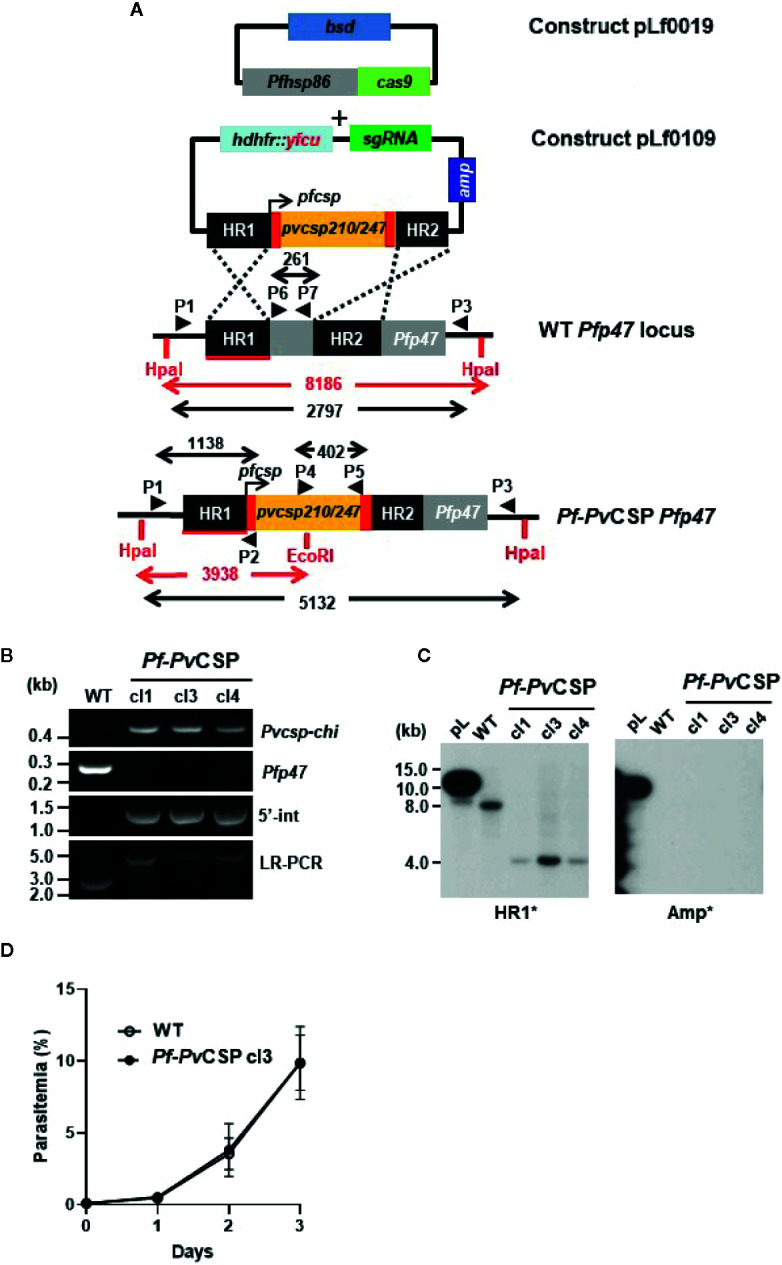
Generation and genotyping of the chimeric *Pf*-*Pv*CSP parasites. A schematic representation of the Cas9 (plasmid Leiden *falciparum* (pLf) 0019) and donor DNA plasmids (pLf0109) constructs used to introduce the *Pvcsp* chimeric (*Pvcsp-chi*) expression cassette into the *Pf*NF54 *p47* gene locus. The *Pvcsp-chi* gene contains a chimeric repeat region of VK210 and VK247 alleles and is under the control of the promoter of the *Pfcsp* gene. The *p47* homology regions (HR1, HR2) used to introduce the donor DNA, location of primers (P), sizes of restriction fragments (*Hpa*I and *Eco*RI; in red), and PCR amplicons (in black) are indicated. WT, wild type; *bsd*, blasticidin selectable marker (bsd); h*dhfr::yfcu*, in donor plasmid. **(B)** Diagnostic PCRs confirming the correct integration of the *Pvcsp-chi* expression cassette into the *Pfp47* locus. Diagnostic PCR: part of *Pvcsp-chi* open reading frame (primers P4/P5); part of *Pfp47* open reading frame (primers P6/P7); 5’-integration of the plasmid into the *Pf*-*Pv*CSP-chi genome (5’-Int; primers P1/P2); LR-PCR (primers P1/P3). **(C)** Southern analysis of *Hpa*I and *Eco*RI restricted DNA of WT, and chimeric *Pf*-*Pv*CSP parasites confirms the specific integration of the *Pvcsp* genes into the *Pfp47* gene locus. **(D)** Growth of asexual blood-stages of *Pf*-*Pv*CSP cl3 and WT *Pf*NF54. Parasitemia (mean and S.D of 3 independent cultures) is shown during a 3-day culture period (in the semi-automated culture system). Cultures were initiated with a parasitemia of 0.1%.

We have previously shown that chimeric *P. falciparum* parasites with the *Pfcsp* gene replaced by the two major *Pvcsp* alleles, VK210 and VK247, do not produce salivary gland sporozoites and most oocysts degenerate before formation of sporozoites (Marin-Mogollon et al., 2018). It has been shown that viable and infectious rodent *P. berghei* sporozoites can be engineered to express CSP proteins from two different *Plasmodium* species. These chimeric sporozoites were generated by introducing a *P. falciparum csp* gene as an additional copy into the *P. berghei* genome (Mendes et al., 2018a; Mendes et al., 2018b; Reuling et al., 2020). Based on this observation we aimed at generating chimeric *P. falciparum* sporozoites expressing both *Pf*CSP and an chimeric *Pv*CSP protein (*Pv*CSP-chi). Using CRISPR/Cas9 gene editing, a chimeric *P. falciparum* line (*Pf*-*Pv*CSP) was created that contains the *Pvcsp-chi* gene under the control of 967 bp of the *Pfcsp* gene promoter region. This expression cassette was introduced into the neutral *Pfp47* locus (*PF*3D7_1346800) of the genome of *P. falciparum* NF54 wild type (WT) parasites. The *Pfp47* locus was selected as it is suitable for introducing transgenes without compromising parasite development in the blood and liver stages or in *A. stephensi* mosquitoes (Talman et al., 2010; Vaughan et al., 2012; Vos et al., 2015; Marin-Mogollon et al., 2019; Miyazaki et al., 2020). The WT *Pf*NF54 parasites were transfected with two plasmids: a previously described construct containing the Cas9 expression cassette with the *blasticidin* (*bsd*) drug-selectable marker cassette (pLf0019) and a donor DNA construct (pLf0109) designed to target the *Pfp47* locus. The donor construct contains the *Pvcsp-chi* expression cassette flanked by two homology regions of *Pfp47*, the *Pfp47* sgRNA sequence and a h*dhfr*-y*fcu* drug-selectable marker cassette (see **Figures 1A**, **S1**, **S2** and *Materials and Methods* section for details of the generation of the constructs). This *Pvcsp-chi* gene contains the N- and C-terminal regions of the *Pvcsp* VK210 allele that flank a chimeric repeat sequence comprising repeats of both the VK210 allele (three times the repeat GDRADGQPA/GDRAAGQPA) and the VK247 allele (three times the repeat ANGAGNQPG/ANGAGDQPG). Transfection of WT *Pf*NF54 parasites was performed using the ‘spontaneous uptake method’. Briefly, uninfected RBC were first transfected with the constructs pLf0019 and pLf0109. Subsequently, the transfected RBCs were mixed with WT *Pf*NF54 infected RBC (iRBC). Selection of transfected parasites containing both plasmids was performed by applying double-positive selection with the drugs Blasticidin and WR99210 until parasites were detectable by thin blood-smear analysis. Subsequently negative drug selection with 5-fluorocytosine was applied to enrich the parasites in which the *Pvcsp-chi* gene expression cassette was integrated into the genome by double cross-over homologous recombination. Next drug-selected parasites were cloned by limiting dilution. Diagnostic (long range) PCR and Southern analyses of three clones (*Pf*-*Pv*CSP cl1, cl3, and cl4) confirmed correct double crossover integration of the *Pvcsp-chi* gene expression into the *Pfp47* locus in the genome of all *Pf*-*Pv*CSP clones (**Figures 1B, C**). Two *Pf*-*Pv*CSP clones (cls 1, 3) were selected for further phenotype characterization.

### Pf-PvCSP Parasites Produce Sporozoites in *Anopheles stephensi* Mosquitoes

The *in vitro* growth of *Pf*-*Pv*CSP (cl3) blood stages was comparable to that of the parental WT *Pf*NF54 blood stage parasites (**Figure 1D**). *Pf*-*Pv*CSP parasites produced numbers of mature gametocytes in standardized gametocyte cultures similar to those of the parental line (**Table 1** **;** **Figure S3**) and male gametocytes underwent exflagellation upon activation. Using SMFA, *A. stephensi* mosquitoes were infected with gametocytes of either WT *Pf*NF54 or *Pf*-*Pv*CSP parasites. The number of oocysts in mosquito midguts was determined at days 10 to 12 after infection and the number of sporozoites in salivary glands was analyzed at days 18 to 24 after infection. These analyses revealed that *Pf*-*Pv*CSP parasites produced comparable number of oocysts and salivary gland sporozoites to those of the parental parasites (**Table 1** **;** **Figures S3** and **S4**). Approximately 90% of *Pf*-*Pv*CSP oocysts already contained sporozoites at days 10 to 12 which is in contrast to the two previously created chimeric *P. falciparum* lines with the *Pfcsp* gene replaced by the *Pvcsp* alleles vk210 and vk247. Most of the oocysts of these replacement lines degenerated before the formation of sporozoites (Marin-Mogollon et al., 2018). The presence of *Pf*-*Pv*CSP sporozoites in salivary glands (**Table 1**) indicated that *Pf*CSP retains its normal function in the formation of sporozoites, and that expression of *Pv*CSP-chi in these sporozoites does not appear to interfere with sporozoite formation and invasion of salivary glands.

**Table 1:** Gametocyte, oocyst, and sporozoite production of *Pf*-*Pv*CSP and WT NF54 lines.

Parasites		Gams (%) male/female mean (SD)^a^	Exfl. No mean (SD)^b^	Exfl. Males (%)Mean (SD)^c^	Infected/dissected mosquitoes	Oocysts No mean (SD)^d^	Spz No mean^e^
**WT** **NF54**	exp. 1	0.45/0.57	366	80	16/18	62	32 K
	exp. 2	0.47/0.67	401	85	9/12	47	21 K
	exp. 3	0.52/0.63 (0.03/0.04)	201 (87)	38 (21)	11/12	40 (9.3)	54 K
***Pf-Pv*CSP**						
**Clone 1**	exp. 1	0.8/1.1	623	76	16/16	44	17 K
**Clone 3**	exp. 1	0.6/1.1	696	100	16/16	63	18 K
****	exp. 2	0.3/0.4	641	100	18/18	52	20 K
	exp. 3	0.7/1.0 (0.2/0.3)	485 (89)	65 (17)	9/9	48 (6.4)	46 K

a Mean percentage of stage V male and female gametocytes (per 100 red blood cells) in day 14 gametocyte cultures in one to three experiments (exp.) with standard deviation (SD).

b Mean number of exflagellating male gametocytes (per 10^5^ red blood cells) at 10–20 min after activation in day 14 gametocyte cultures.

c Mean percentage of exflagellating stage V male gametocytes in day 14 gametocyte cultures (1–3 exp.) with standard deviation (SD).

d Mean number of oocyst per mosquito at days 10 to 12 after feeding (1–3 exp. per line; 10–30 mosquitoes per exp.).

e Mean number of salivary gland sporozoites per mosquito at days 18–24 after feeding. Range corresponds to the mean number of sporozoites in multiple experiments (1–3 exp. per line; 50–90 mosquitoes per exp.). See **Figure S3** for a graphic representation of the data of independent experiments.

### Pf-PvCSP Sporozoites Express PfCSP As Well As PvCSP-chi on Their Surface

**Figure 2:**
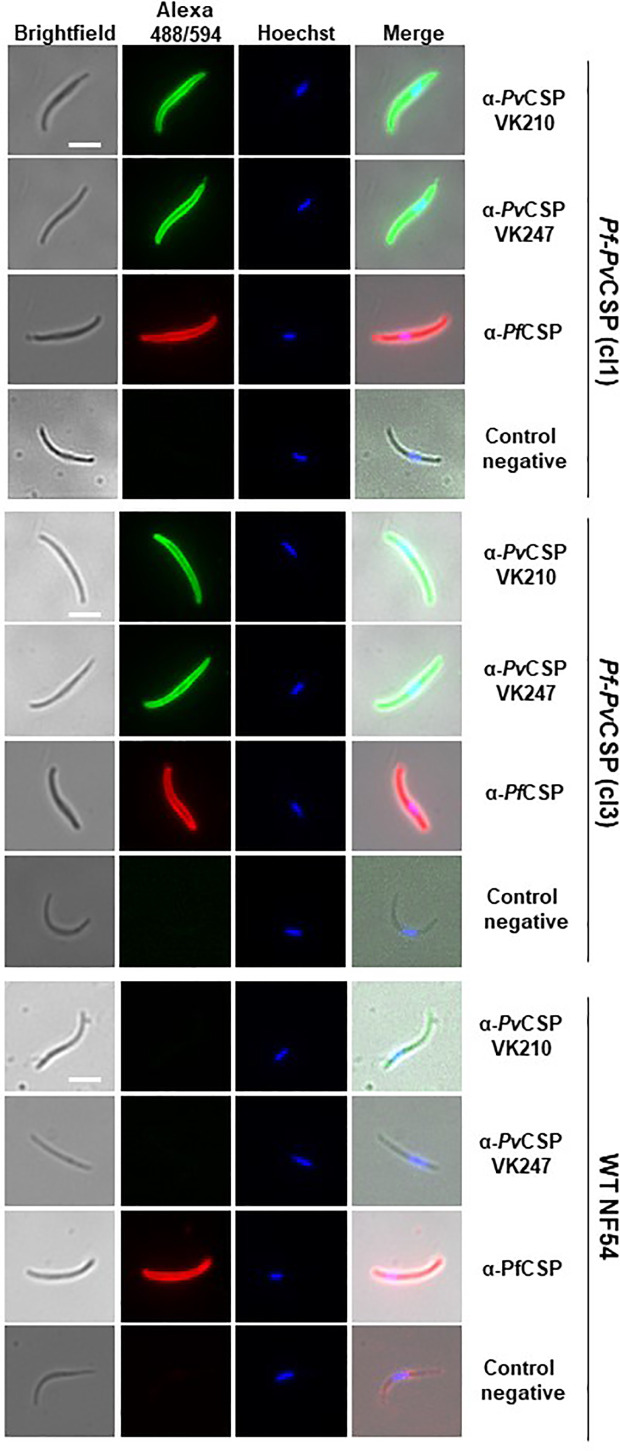
Immunofluorescence analyses of *Pv*CSP and *Pf*CSP expression in fixed *Pf*-*Pv*CSP salivary gland sporozoites. Fixed permeabilized sporozoites of *Pf*-*Pv*CSP (clone 1 and clone 3) and WT *Pf*NF54 parasites stained with mouse anti-*Pv*CSPVK210, anti-*Pv*CSP VK247 (green; alexa 488) and anti-*Pf*CSP antibodies (red; alexa 594). Nuclei stained with the Hoechst-33342. Control negative, corresponds to the incubation without the primary antibody (2 experiments). Scale bar is 5 µm.

To analyze the expression of *Pv*CSP-chi and *Pf*CSP in salivary gland sporozoites of the *Pf*-*Pv*CSP line, we first performed an immunofluorescence on fixed/permeabilized sporozoites with antibodies specific for the repeat sequences of either *Pv*CSP VK210, *Pv*CSP VK247 or *Pf*CSP. All *Pf*-*Pv*CSP sporozoites analyzed (n=2 exp.) reacted with all three antibodies whereas WT *Pf*NF54 sporozoites reacted only with anti-*Pf*CSP antibodies (**Figure 2**), indicating that all *Pf*-*Pv*CSP sporozoites expressed both the *Pf*CSP and *Pv*CSP-chi proteins.

**Figure 3:**
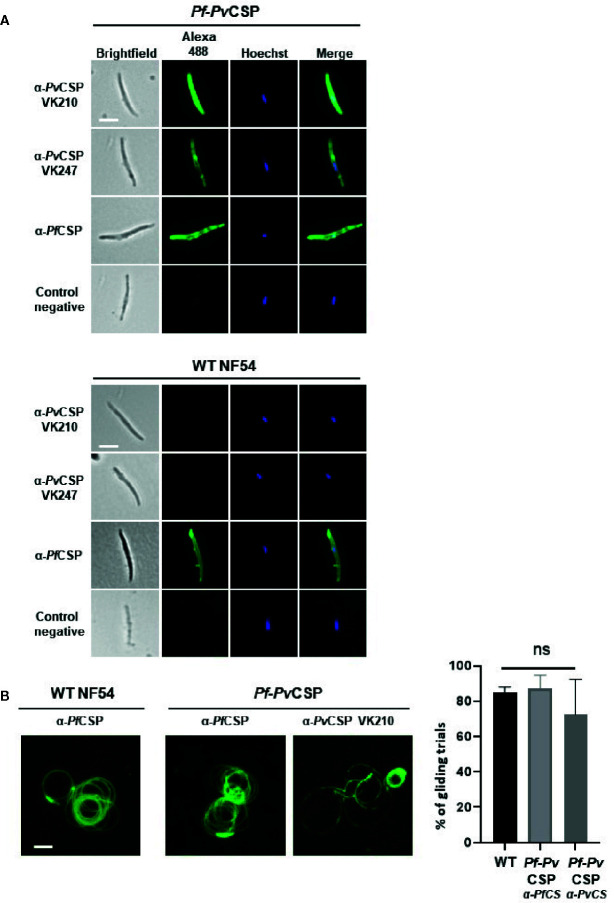
Immunofluorescence analyses of *Pv*CSP and *Pf*CSP expression of live *Pf*-*Pv*CSP salivary gland sporozoites. **(A)** Live sporozoites of *Pf*-*Pv*CSP and WT *Pf*NF54 parasites labelled with mouse anti-*Pv*CSPVK210, anti-*Pv*CSP VK247 and anti-*Pf*CSP antibodies (green; alexa 488). Nuclei stained with the Hoechst-33342. Control negative, corresponds to the incubation without the primary antibody (2 experiments). Scale bar is 5 µm. **(B)** Typical sporozoite gliding trails of *Pf*-*Pv*CSP and WT *Pf*NF54 sporozoites stained with mouse anti-*Pv*CSPVK210 and anti-*Pf*CSP antibodies (left) and percentage of gliding trials (right) using primary antibody anti-Pf-3SP2 and anti-*Pv*CSPVK210 (1 experiment). Scale bar is 15 µm. ns, not significant, as determined by One-way ANOVA test (GraphPad).

We next investigated whether *Pv*CSP-chi protein is expressed at the surface of sporozoites, by performing IFA of live salivary gland sporozoites. Similarly to the results of IFA using fixed/permeabilized sporozoites, live *Pf*-*Pv*CSP sporozoites reacted with anti-*Pv*CSP VK210, anti-*Pv*CSP VK247 and anti-*Pf*CSP antibodies whereas WT *Pf*NF54 sporozoites reacted only to anti-*Pf*CSP antibodies (**Figure 3A**). This result demonstrates that the *Pv*CSP-chi protein is expressed at the surface of *Pf*-*Pv*CSP sporozoites.

As CSP is shed during parasite gliding motility, we next assessed if *Pv*CSP is also shed similarly to *Pf*CSP by *Pf*-*Pv*CSP sporozoites. CSP-trails in a sporozoite gliding assay also reacted with both anti-*Pf*CSP and anti *Pv*CSP VK210 antibodies (**Figure 3B**), confirming surface expression of *Pv*CSP-chi and revealing shedding of *Pv*CSP-chi during gliding. Comparable gliding trails were observed in WT *Pf*NF54 and *Pf*-*Pv*CSP sporozoites using anti-*Pf*CSP antibody (**Figure 3B**), with a percentage of 85% gliding trails in WT *Pf*NF54 sporozoites (with a range of 28–65 gliding trails per field, Stdev. 2.6) and 87.0% gliding trails in *Pf*-*Pv*CSP sporozoites (with a range of 23–43 gliding trails per field, Stdev 7,6.).

### Pf-PvCSP Sporozoites Infect Primary Human Hepatocytes

**Figure 4:**
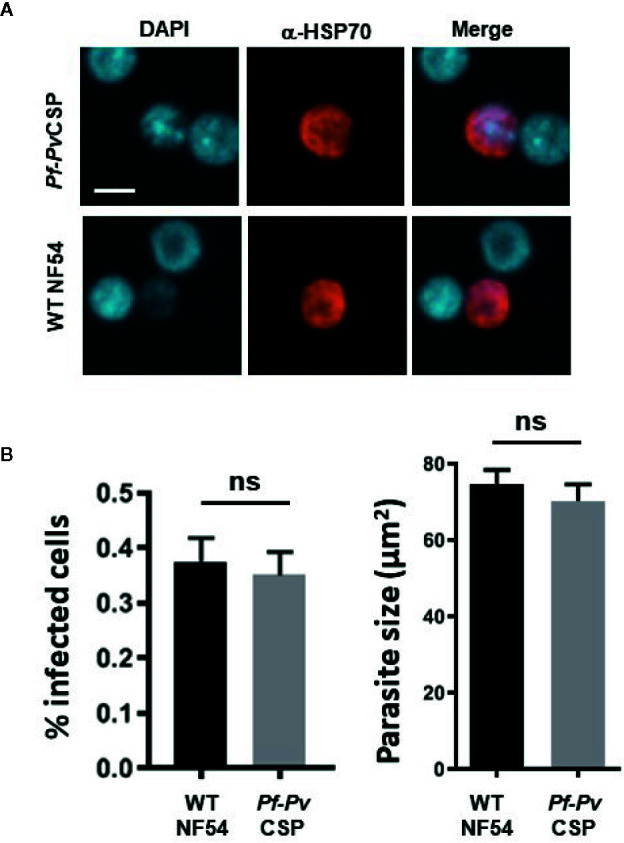
*In vitro* infection of primary human hepatocytes by *Pf*-*Pv*CSP sporozoites. **(A)** Representative images of liver stage development in primary human hepatocytes 4 days after infection with *Pf*-*Pv*CSP and WT *Pf*NF54 sporozoites. Liver stages were stained with anti-*Pf*HSP70 antibodies and nuclei were stained with DAPI (1 experiment). Scale bar is 10 µm. **(B)** Percentage of infected hepatocytes (left) and size of liver stages (right) at day 4 after infection of hepatocytes by *Pf*-*Pv*CSP and WT NF54 sporozoites. The size was measured by determining the area of the parasite cytoplasm using the red-positive (anti-HSP70) area (µm^2^). n.s., not significant, as determined by unpaired t-test (GraphPad).

We next assessed the *in vitro* infectivity of *Pf*-*Pv*CSP sporozoites to primary human hepatocytes. At day 4 post infection of primary human hepatocytes with either WT or *Pf*-*Pv*CSP sporozoites, the cells were fixed, and liver stages were stained with an anti-*Pf*HSP70 antibody. *Pf*-*Pv*CSP sporozoites showed comparable efficiency of hepatocyte infection to WT *Pf*NF54 sporozoites (**Figure 4**). *Pf*-*Pv*CSP and WT *Pf*NF54 also displayed similar liver stage sizes at day 4 post infection (**Figure 4**). These results demonstrate that expression of *Pv*CSP-chi at the surface of *Pf*-*Pv*CSP sporozoites did not reduce parasite *in vitro* invasion and development in primary human hepatocytes.

#### Immunization of Mice with Pf-PvCSP Sporozoites Induce Antibodies Against Repeats of Both PfCSP and PvCSP-chi

**Figure 5:**
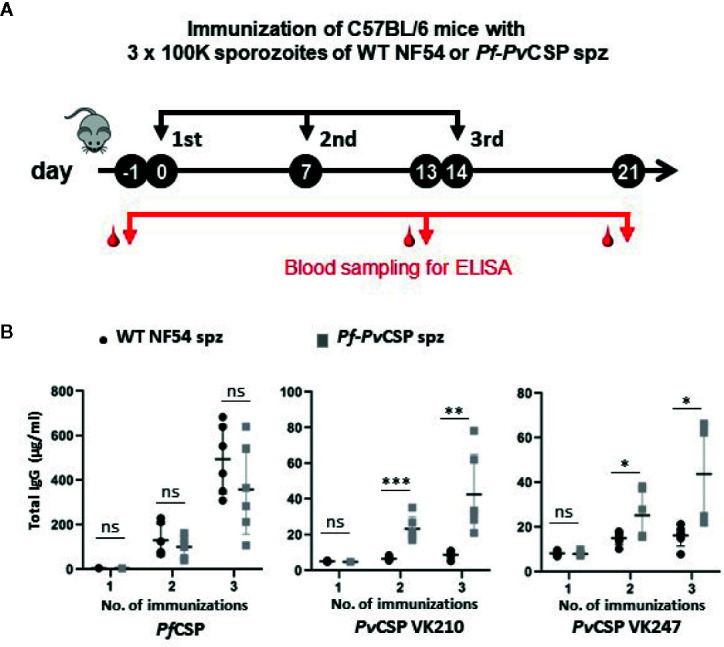
Antibody production against repeat sequences of PfCSP and PvCSP-chi in mice after immunization with *Pf*-*Pv*CSP and WT *Pf*NF54 sporozoites. **(A)** Schematic representation of the immunization protocol. Groups of six C57BL/6 mice were immunized three times with killed sporozoites with a 7-day interval and blood was collected from all mice by orbital vein puncture 1 day before the first and third immunization and one week after the last immunization (one experiment). **(B)** Total IgG titers against *Pf*CSP and *Pv*CSP repeat sequences in plasma prior, during and after immunization. Enzyme-linked immunosorbent assays were performed with plasma samples collected from mice as shown in **(A)** to assess total IgG titers specific for the repeat sequences of *Pf*CSP ((NANP)4NVDPC) and two variants of *Pv*CSP(GD RAD GQP AGD RAA GQP A for *Pv*CSP VK210; ANGAGNQPG ANGAGDQPG for the *Pv*CSP VK247. Mean and standard error of the mean and *p < 0.05; **p < 0.01; ***p < 0.001, as determined by unpaired t-test analysis.

We then analyzed the capacity of the *Pf*-*Pv*CSP sporozoites to elicit the production of antibodies against the repeat sequences of *Pf*CSP and *Pv*CSP-chi by immunizing mice with killed *Pf*-*Pv*CSP and WT *Pf*NF54 sporozoites. Groups of six C57BL/6 mice were intravenously injected with 10^5^ freeze-thaw killed sporozoites, three times with a 7-day interval, and blood was collected from all mice 1 day prior to the first and third immunization and 1 week after the last immunization (**Figure 5A**). Plasma of these mice was tested for antigen-specific antibody production by ELISA using plates that were coated with the following repeat sequences; (NANP)4NVDPC for *Pf*CSP; GD RAD GQP AGD RAA GQP A for *Pv*CSP VK210; and ANGAGNQPG ANGAGDQPG for *Pv*CSP VK247 strain.

Plasma samples of mice immunized with either WT *Pf*NF54 or *Pf*-*Pv*CSP sporozoites reacted strongly to the *Pf*CSP repeat sequences after the second and third immunization with no significant differences in total IgG between the two groups of mice (**Figure 5B**), indicating comparable immunogenicity of the *Pf*CSP repeat sequences in WT *Pf*NF54 or *Pf*-*Pv*CSP sporozoites. In contrast, only mice immunized with *Pf*-*Pv*CSP sporozoites showed significantly increased antibody titers specific for the repeat sequences of *Pv*CSP VK210 and *Pv*CSP VK247 whereas no significant increase was observed in WT *Pf*NF54-immunized mice (**Figure 5B**). Although the total IgG against the *Pv*CSP repeats was lower than that against the *Pf*CSP repeats, these results show that exposure to *Pf*-*Pv*CSP sporozoites can induce the production of antibodies against *Pf*CSP, as well as against both *Pv*CSP repeat variants.

## Discussion

In this study we describe a chimeric *P. falciparum* line, *Pf*-*Pv*CSP, which expresses CSP from both *P. falciparum* and *P. vivax*. We demonstrate that chimeric parasites produce sporozoites in similar numbers to those of the parental WT *Pf*NF54 line and that both CSPs expressed in the chimeric parasites are present at the sporozoite surface and shed during gliding. Our results show that *Pf*-*Pv*CSP sporozoites are infectious to primary human hepatocytes. These observations support the notion that the presence of the chimeric *Pv*CSP protein at the surface of *P. falciparum* sporozoites does not affect sporozoite viability and infectivity. The formation of infective sporozoites expressing CSP of two different *Plasmodium* species has been previously demonstrated in studies using *P. berghei* expressing *Pf*CSP (Mendes et al., 2018b). However, the viability and infectivity of these chimeric *Pb-PfCSP* sporozoites is expected, since *Pf*CSP can fully complement the multiple functions of *Pb*CSP as shown in replacement *P. berghei* lines with the endogenous *Pb*CSP replaced by *Pf*CSP (Triller et al., 2017; Flores-Garcia et al., 2019). This is distinct from *Pv*CSP, since previous attempts to functionally complement *Pf*CSP, by replacing the *Pfcsp* gene with two *Pvcsp alleles*, were unsuccessful (Marin-Mogollon et al., 2018). It is known that CSP fulfills multiple functions, both in the process of sporozoite formation and in later processes of sporozoite invasion of salivary glands, migration through the human skin and invasion of hepatocytes (Sinnis and Coppi, 2007; Vaughan et al., 2008; Ejigiri and Sinnis, 2009; Coppi et al., 2011; Vaughan and Kappe, 2017).

Although the previously generated gene-replacement *Pf-PvCSP* parasites were able to produce normal numbers of oocysts, no salivary gland sporozoite were formed and most oocyst degenerated before sporozoite formation, a phenotype that is comparable to *P. falciparum* mutants lacking the *Pfcsp* gene (Marin-Mogollon et al., 2018). Collectively, these observations indicate that *Pv*CSP is unable to fully complement the role of *Pf*CSP in the process of sporozoite formation inside oocysts but that the simultaneous expression of *Pv*CSP with *Pf*CSP in sporozoites of chimeric parasites does not hamper the formation of infectious sporozoites. The lack of full complementation of *Pf*CSP by *Pv*CSP in the process of sporozoite formation may result from the inability of *Pv*CSP to interact with other *P. falciparum* proteins that are involved in (the initial steps of) sporozoite formation.

Our studies of immunization of mice with the chimeric *Pf-PvCSP* sporozoites shows that antibodies are induced against the repeat sequences of both *Pf*CSP and the *Pv*CSP-chi protein, including antibody responses against both *Pv*CSP alleles, VK210 and VK247. Interestingly, although IgG responses against *Pf*CSP were significantly higher than against *Pv*CSP, anti- *Pf*CSP titers were comparable for mice immunized with either WT *Pf*NF54 sporozoites or *Pf-PvCSP* sporozoites. These observations indicate that the presence of *Pv*CSP does not affect the immunogenicity of *Pf*CSP with respect to IgG responses against the NANP/NVDP repeats. The lower IgG responses against the two *Pv*CSP repeats compared to the *Pf*CSP NANP/NVDP repeats can be caused by different factors. The *Pv*CSP-chi protein contains only 3 copies of the VK247 and VK210 repeats, while the *P. falciparum* NF54 CSP protein contains 38 NANP and 4 NVDP repeats (Bowman et al., 1999; Oyen et al., 2018). In addition, although we provide evidence of the presence of *Pv*CSP at the surface of the *Pf-Pv*CSP sporozoites and in gliding trails, the total amount of *Pf*CSP and *Pv*CSP in the *Pf-Pv*CSP sporozoites is unknown and may differ for the two proteins. The lower IgG responses might also result from a lower intrinsic immunogenicity of the *Pv*CSP repeats compared to the *P. falciparum* NANP/NVDP repeats. The repeat regions of CSP of the *P. vivax* strains VK210 and VK247 consist of 10–11 copies of the repeats GDRA(A/D)GQPA or ANGA (G/D)(N/D)QPG, respectively (de Souza-Neiras et al., 2007)] and are not cross reactive (Hall et al., 2019). Of note, our study employed freeze-thaw killed sporozoites to immunize mice; it is therefore possible that antibody responses against *Pf*CSP and *Pv*CSP-chi proteins might be different when immunization is performed with live sporozoites.

Despite the differences in the repeat regions of *P. falciparum* and *P. vivax* CSP, both these proteins have been shown to be immunogenic and antibody levels against these repeats appear to be associated with protective immunity in animal malaria models and in humans (Porter et al., 2013; Foquet et al., 2014; Schwenk et al., 2014; Yadava et al., 2014; White et al., 2015; Gimenez et al., 2017; Atcheson et al., 2018; de Camargo et al., 2018; Dobaño et al., 2019). However, due to differences in methodologies for inducing and analyzing antibody production after immunization with full-length CSP proteins or the respective repeat sequences, the relative immunogenicity of the *Pf*CSP and *Pv*CSP repeats in both animal models and in humans is largely unknown. To our knowledge, no studies exist that have compared the immunogenicity of different CSP repeat sequences of the two human *Plasmodium* species using similar immunization approaches and delivery systems. In comparative *P. vivax* and *P. falciparum* CHMI studies, it has been found that the CSP of both species induced cross-reactive antibodies (Hall et al., 2019). However, it is unknown whether the observed cross-reactivity resulted from the repeat sequences or from other epitopes in the N- or C-terminal region of the CSP molecules. In future studies it will be relevant to analyze infections in the FRG huHep mouse model (repopulated with human RBCs) to show that *Pf-Pv*CSP can form infectious liver merozoites. This would enable the use of this model for testing the efficacy of anti-*Pv*CSP antibodies.

Combined, our observations regarding the expression and immunogenicity of the *Pv*CSP on the surface of *P. falciparum* sporozoites hold promise for the use of the chimeric *Pf*-*Pv*CSP sporozoites for clinical evaluation of vaccines targeting *Pv*CSP in clinical CHMI studies. The use of such parasites would bypass the need for *P. vivax* sporozoite production and measures for hypnozoite removal (Payne et al., 2017). In addition, such chimeric sporozoites might also be used in whole, attenuated sporozoite vaccination approaches (Bijker et al., 2015; Goswami et al., 2020). Vaccination with chimeric, attenuated sporozoites might induce cross-protective immune responses against both *P. falciparum* and *P. vivax*.

## Author’s Note

This paper is dedicated to the memory of our friend and colleague, SMK, who initiated the studies described in this paper and recently passed away.

## Data Availability Statement

The raw data supporting the conclusions of this article will be made available by the authors, without undue reservation.

## Ethics Statement

The animal study was reviewed and approved by Animal Experiments Committee Leiden (AVD1160020171625). All experiments were performed in accordance with the Experiments on Animals Act (Wod, 2014), the applicable legislation in the Netherlands and in accordance with the European guidelines (EU directive no. 2010/63/EU) regarding the protection of animals used for scientific purposes.

## Author Contributions

CM-M, BF-F, CJ, and SMK came up with the study concept and design. YM, CM-M, TI, AM, RL, AS, FG, SM, SC-M, JR, SK, HK, RS, AR-S, MP, CJ, and BF-F acquired the data. YM, CM-M, AM, MP, CJ, and BF-F conducted analysis and interpretation of the data. YM, CM-M, BF-F, and CJ wrote the draft of the manuscript. YM, CM-M, SM, AM, MP, RS, CJ, and BF-F critically revised the manuscript for important intellectual content. AM, KD, BW, and RS provided technical and/or material support. BF-F and CJ supervised the study. All authors reviewed the manuscript. All authors contributed to the article and approved the submitted version.

## Funding

CM-M was, in part, supported by Colciencias Ph.D. fellowship (Call 568 from 2012 Resolution 01218 Bogotá, Colombia). TI was, in part, supported by Uehara Memorial Foundation grant. Work performed at IMM was supported by Fundação para a Ciência e Tecnologia (FCT-Portugal)’s grants PTDC/BBB-BMD/2695/2014 and PTDC-SAU-INF-29550-2017. AR-S is supported by the MRC-DPFS grant MR/N019008/1.

## Conflict of Interest

KD holds stock in TropIQ Health Sciences. RL, AS and KD were employed by TropIQ Health Sciences.

The remaining authors declare that the research was conducted in the absence of any commercial or financial relationships that could be construed as a potential conflict of interest.

## Supplementary Material

Supplementary Figure 1 Plasmid maps of plasmids used in this study.

Supplementary Figure 2 Plasmid map of pLf0109 and sequence of the commercially synthesized *Pvcsp-chi* gene. This gene that contains a codon-optimized *Pvcsp* open reading frame containing the N- and C-terminal regions of the *Pv*csp VK210 allele that flank a chimeric repeat sequence comprising repeats of both the VK210 allele (three times the repeat GDRADGQPA/GDRAAGQPA) and the VK247 allele (three times the repeat ANGAGNQPG/ANGAGDQPG).

Supplementary Figure 3 Percentage of male exflagellating gametocytes and number of oocyst and sporozoites of *Pf*-*Pv*CSP cl3 and WT *Pf*NF54 parasites. **(A)** Percentage of stage V exflagellating male gametocytes in day 14 gametocyte cultures (3 experiments). Each dot correspond to an independent experiment. **(B)** Number of oocyst per mosquito at day 10 after feeding (n=3). Each dot correspond to the number of oocysts per midgut. **(C)** Number of sporozoites per mosquito at day 18–24 after feeding (n=3). Each dot corresponds to the mean number of sporozoites per mosquito. Mean and standard error of the mean. n.s., not significant (unpaired t-test; Graphpad).

Supplementary Figure 4 Sporozoite formation in oocysts of *Pf*-*Pv*CSP and WT *Pf*NF54 parasites. Representative images of oocyst during development **(A)** and sporozoite formation **(B)** in oocysts (day 10) from WT NF54 parasites and *Pf-Pv*CSP parasites that express both *Pf*CSP and *Pv*CSP-chi. **(A)** Complete midgut and magnified area (×5). Scale bar: 200 µm (upper row) and 20 µm (lower row). **(B)** Sporozoite formation in an unstained oocyst. Scale bar: 20 µm.

Supplementary Table 1 Primers used in this study.
