# Supplementary figures and images for: Generation of a Genetically Modified Chimeric Plasmodium falciparum Parasite Expressing Plasmodium vivax Circumsporozoite Protein for Malaria Vaccine Development
Source: Front Cell Infect Microbiol. 2020 Dec 17;10:591046. doi: 10.3389/fcimb.2020.591046 (PMC7773900; doi:10.3389/fcimb.2020.591046)

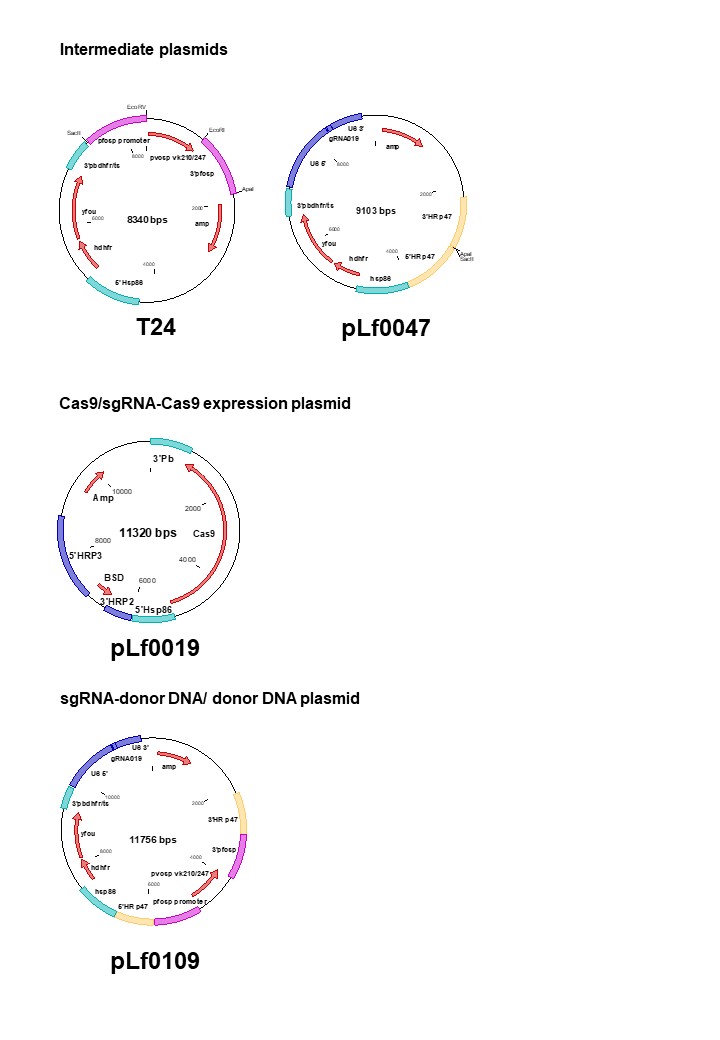

Supplement: Supplementary Figure 1 — Plasmid maps of plasmids used in this study. [file Image_1.jpeg]

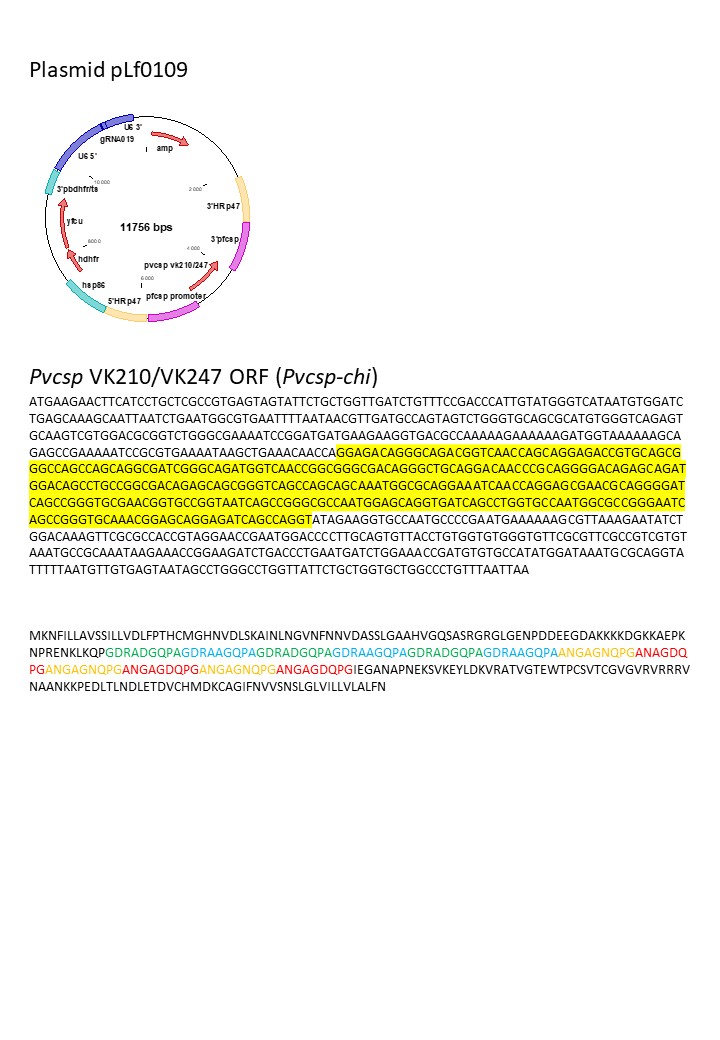

Supplement: Supplementary Figure 2 — Plasmid map of pLf0109 and sequence of the commercially synthesized Pvcsp-chi gene. This gene that contains a codon-optimized Pvcsp open reading frame containing the N- and C-terminal regions of the Pvcsp VK210 allele that flank a chimeric repeat sequence comprising repeats of both the VK210 allele (three times the repeat GDRADGQPA/GDRAAGQPA) and the VK247 allele (three times the repeat ANGAGNQPG/ANGAGDQPG). [file Image_2.jpeg]

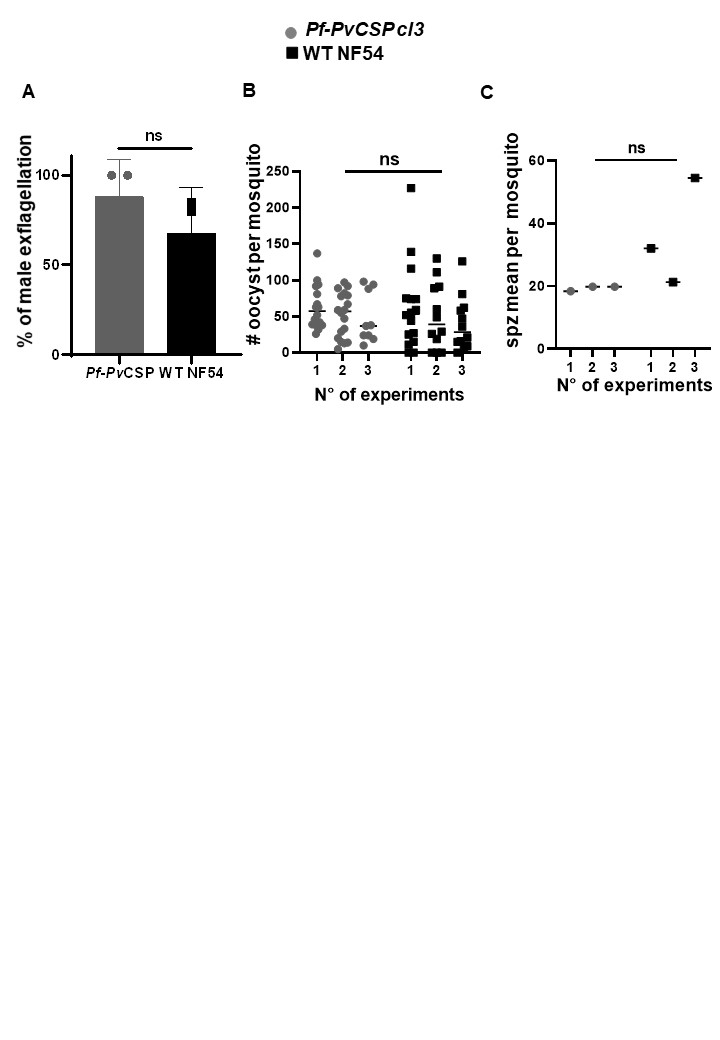

Supplement: Supplementary Figure 3 — Percentage of male exflagellating gametocytes and number of oocyst and sporozoites of Pf-PvCSP cl3 and WT PfNF54 parasites. (A) Percentage of stage V exflagellating male gametocytes in day 14 gametocyte cultures (3 experiments). Each dot correspond to an independent experiment. (B) Number of oocyst per mosquito at day 10 after feeding (n=3). Each dot correspond to the number of oocysts per midgut. (C) Number of sporozoites per mosquito at day 18–24 after feeding (n=3). Each dot corresponds to the mean number of sporozoites per mosquito. Mean and standard error of the mean. n.s., not significant (unpaired t-test; Graphpad). [file Image_3.jpeg]

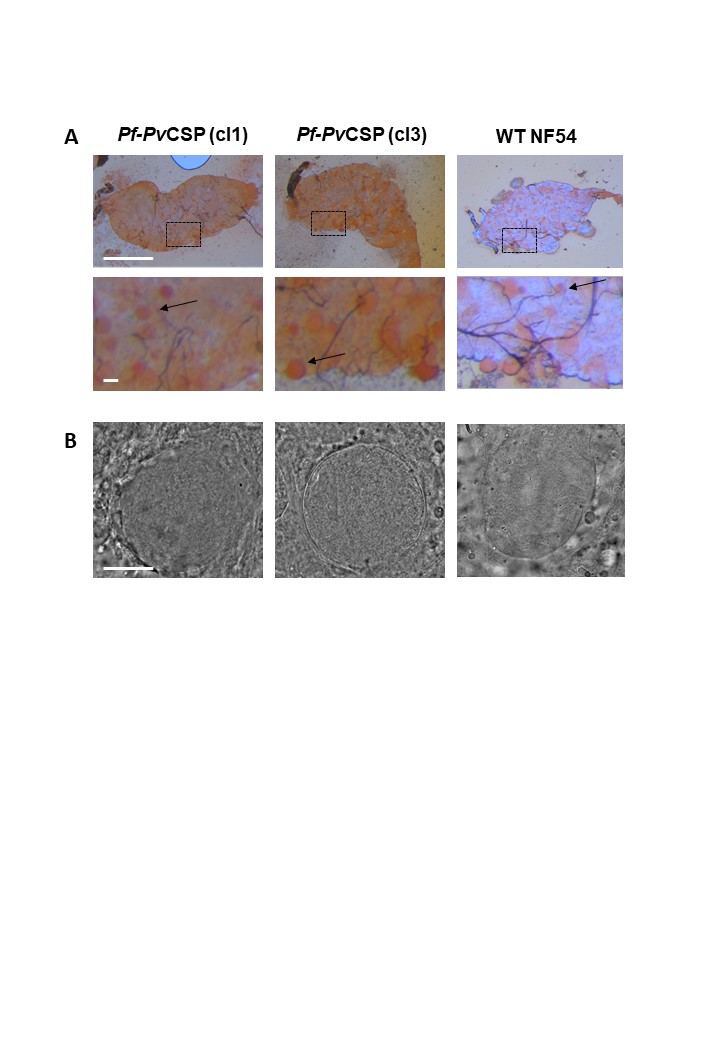

Supplement: Supplementary Figure 4 — Sporozoite formation in oocysts of Pf-PvCSP and WT PfNF54 parasites. Representative images of oocyst during development (A) and sporozoite formation (B) in oocysts (day 10) from WT NF54 parasites and Pf-PvCSP parasites that express both PfCSP and PvCSP-chi. (A) Complete midgut and magnified area (×5). Scale bar: 200 µm (upper row) and 20 µm (lower row). (B) Sporozoite formation in an unstained oocyst. Scale bar: 20 µm. [file Image_4.jpeg]
